# *In vitro* antibacterial activity of combinations of fosfomycin, minocycline and polymyxin B on pan-drug-resistant *Acinetobacter baumannii*

**DOI:** 10.3892/etm.2013.1039

**Published:** 2013-04-02

**Authors:** YAJUN ZHANG, FENGZHE CHEN, ENHUA SUN, RUIPING MA, CHUNMEI QU, LIXIAN MA

**Affiliations:** 1 Departments of Infectious Diseases, Shandong 250012, P.R. China; 2 Laboratory Medicine, Qilu Hospital, Shandong University, Jinan, Shandong 250012, P.R. China

**Keywords:** pan-drug resistant *Acinetobacter baumannii*, minocycline, fosfomycin, polymyxin B, minimum inhibitory concentration, fractional inhibitory concentration index

## Abstract

The aim of this study was to determine the effects of combinations of fosfomycin, minocycline and polymyxin B in the treatment of pan-drug-resistant *Acinetobacter baumannii* (PDR-Ab). The *in vitro* antibacterial activities of the drugs were evaluated by determination of the minimum inhibitory concentration (MIC) and the fractional inhibitory concentration index (FICI). A total of 25 strains of PDR-Ab were selected using the VITEK32 microbial analysis instrument and the Kirby-Bauer (K-B) method. A broth microdilution method was used to determine the MIC for each of the three drugs, and the checkerboard method was simultaneously used to determine the MICs for combinations of the drugs. FICI values were also calculated. While fosfomycin alone was ineffective for the treatment of PDR-Ab, its MIC value was significantly reduced when used in combination with minocycline or polymyxin B. The combined use of minocycline and polymyxin B also significantly reduced the MIC value of each drug. The FICI values revealed that the drugs had synergistic or additive effects when used in combination. The determination of the MIC and FICI values for the combinations of drugs demonstrated that there is synergistic or additive effect upon the combined use of fosfomycin with minocycline or polymyxin B. The combined use of minocycline and polymyxin B also results in a significant reduction in the MIC values of the two drugs. These experimental results may provide a basis for the future clinical treatment of *Acinetobacter baumannii*.

## Introduction

Over the past decade, the emergence of multi-and even pan-drug-resistant *Acinetobacter baumannii* (PDR-Ab) has brought a tremendous challenge to hospital infection control and clinical treatment. As an opportunistic pathogen widely distributed in the hospital and the natural environment, *Acinetobacter baumannii* is normally colonized in the respiratory, digestive, urinary and reproductive systems. It causes nosocomial infections such as sepsis, ventilator-associated pneumonia and urinary tract infections under certain conditions (1). In recent years, the extensive use and misuse of antibiotics have led to the increasing resistance of *Acinetobacter baumannii*, which has attracted significant attention from relevant personnel.

Drugs that are potentially effective for the treatment of multi-resistant *Acinetobacter baumannii* include carbapenems, tetracyclines and enzyme inhibitors, and polymyxins (1). Polymyxins are regarded as the last line of defense. However, a single medication is typically ineffective in clinical treatment (1). Therefore, while it remains necessary to develop new drugs, at present, the only method for the treatment of *Acinetobacter baumannii* infection is the novel utilization of traditional drugs or the combined use of multiple drugs (2).

In the current study, we examined the effects of combinations of fosfomycin (3–5), minocycline (6) and polymyxin B (7) in the treatment of PDR-Ab. The three drugs were chosen as they show considerable effectiveness and are commonly used in combination in the treatment of multi-resistant bacteria. Our results suggest that the effects of multiple drugs used in combination are synergistic and additive, particularly for the combined use of polymyxin B and minocycline.

## Materials and methods

### Experimental strains

A total of 25 strains of PDR-Ab were collected from the Qilu Hospital of Shandong University (Jinan, China). They were identified to be resistant to multiple drugs, including carbapenems, quinolones, cephalosporins, aminoglycosides and sulfonamides, by the Kirby-Bauer (K-B) method. Among them, 24 strains were from sputum specimens and one was from wound secretions. The strains were stored at −80°C. *Escherichia coli* ATCC25922 was used as a quality control strain.

### Materials and instruments

Minocycline and fosfomycin were purchased from the National Institute for the Control of Pharmaceutical and Biological Products (Beijing, China). Polymyxin B in this study was INALCO1758-9325 (Baierdi Biotechnology Company, Beijing, China). MH agar and MH broth were purchased from Boshang Biotechnology Company (Jinan, China). The VITEK32 microbial analysis instrument was purchased from Boshang Biotechnology Company.

### Broth microdilution method

Bacterial suspensions were prepared by inoculation of colonies from a freshly cultured plate, followed by culturing for 4–6 h at 35°C. The turbidity of the cultures was then calibrated to 0.5 McFarland (1.5×10^8^ CFU/ml) using a spectrophotometer. Stock solutions of antibiotics were prepared and stored at −60°C.

For the determination of the minimum inhibitory concentration (MIC) value of each drug, various concentrations of the drugs were added to a 96-well plate. Bacterial suspensions were added to each well at a final concentration of 1.5×10^5^ CFU/ml and incubated at 35±2°C for 18–24 h. The MIC value was determined as the drug concentration at which bacterial growth was completely inhibited.

For the joint drug susceptibility test, the fold drug dilutions were determined according to the MIC values of each drug. Each combination of two drugs at various concentrations was mixed with a bacterial suspension at a final concentration of 1.5×10^5^ CFU/ml, and incubated at 35±2°C for 18–24 h. The MIC values of each drug were recorded. The fractional inhibitory concentration index (FICI) was calculated as follows: FICI = MIC_A2_/MIC_A1_ + MIC_B2_/MIC_B1_, where MIC_A2_, the MIC value of drug A in combinative use; MIC_A1_, the MIC value of drug A used alone; MIC_B2_, the MIC value of drug B in combinative use; and MIC_B1_, the MIC value of drug B used alone. FICI values of ≤0.5, 0.5–1.0, 1.0–4.0 and >4.0 were considered to indicate a synergistic effect, additive effect, independent effect and antagonistic effect, respectively (8).

### Statistical analysis

SPSS 17.0 software (SPSS, Inc., Chicago, IL, USA) was used to perform the statistical analysis, using a paired t-test and the geometric mean. P<0.05 was considered to indicate a statistically significant result.

## Results

### MIC values are significantly reduced when fosfomycin is used in combination with minocycline or polymyxin B

**Figure 1:**
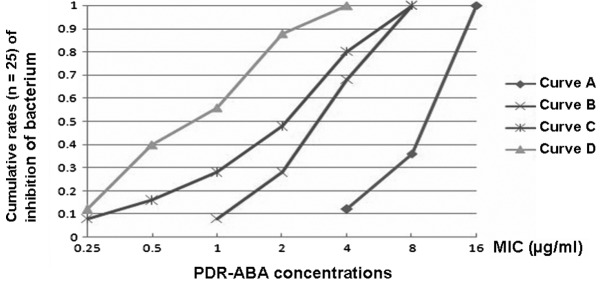
Antibacterial effects of minocycline, polymyxin B and their combinations. Curve A, concentrations of minocycline when used alone; curve B, concentrations of polymyxin B when used alone; curve C, concentrations of minocycline when used in combination with polymyxin B; curve D, concentrations of polymyxin B when used in combination with minocycline. PDR-ABA, pan-drug-resistant *Acinetobacter baumannii*; MIC, minimum inhibitory concentration.

As shown in Tables I and II, the MIC values of fosfomycin, minocycline and polymyxin B when used in combination were significantly reduced when compared with those when used alone. Notably, while fosfomycin alone showed no significant antibacterial effects on *Acinetobacter baumannii*, its MIC value was significantly reduced when used in combination with minocycline or polymyxin B (Tables I and II). We further analyzed antibacterial effects for combinations of minocycline and polymyxin B. The results in Fig. 1 indicate that the MIC values of minocycline and polymyxin B when used in combination were significantly reduced when compared with those when used alone (Fig. 1).

### Effects of combinations of drugs

As demonstrated in Table III, the FICI values for combinations of minocycline and polymyxin B were generally ≤0.5 or >0.5–1.0, suggesting that the two drugs have a synergistic or additive effect. However, the FICI values for fosfomycin and polymyxin B were mostly within the ranges 0.5–1.0 and 1.0–4.0, suggesting that the effects of fosfomycin and polymyxin B were additive or independent. Similar results were also observed for fosfomycin and minocycline. No antagonistic effects for the drug combinations were observed. Based on these findings, the drugs when used in combination have synergistic, additive or independent effects, rather than antagonistic effects. The synergistic and additive effects were the most prominent, particularly for the combination of minocycline and polymyxin B.

## Discussion

In the treatment of infections caused by pan-drug-resistant bacteria, a single medication is typically ineffective. Therefore, the combined use of multiple drugs is recommended. The medication regimen is usually designed by referring to the joint drug susceptibility test *in vitro*. For the evaluation of the combined effect of antimicrobial agents, the FICI value is an important parameter (9). It is considered that the combined use of drugs with a synergistic or additive effect as determined by the FICI is likely to be effective in clinical treatments.

In the current study, we examined the effects of combinations of fosfomycin, minocycline and polymyxin B in the treatment of PDR-Ab. Our results revealed that the MIC values of the drugs were reduced when they were used in combination, suggesting a synergistic or additive effect. This effect was further demonstrated by determining the FICI values.

Based on these findings, the combined use of multiple drugs is effective, which provides a basis for the use of drug combinations in the clinical treatment of PDR-Ab. Furthermore, it also has other advantages over the traditional use of a single medication. By using combinations of multiple drugs, bacterial resistance is likely to be reduced. The incidence of adverse drug responses is also likely to be reduced due to the use of lower drug doses.

## Figures and Tables

**Table I:** Antimicrobial MIC_G_, MIC_50_, and MIC_90_ values of fosfomycin, minocycline and polymyxin B (*μ*g/ml).

Antimicrobial drugs	MIC_range_	MIC_G_	MIC_50_	MIC_90_
Polymyxin B	1–8	3.8906	4	8
Minocycline	4–16	11.4716	16	16
Fosfomycin	>512	>512	-	-

MIC_range_, the range of the MIC values; MIC_G_, the mean MIC value; MIC_50_, the MIC value at which 50% of bacteria were inhibited; MIC_90_, the MIC value at which 90% of bacteria were inhibited.

**Table II:** MIC values of fosfomycin, minocycline and polymyxin B in combined use (*μ*g/ml).

Combined medication	MIC_range_	MIC_G_	MIC_50_	MIC_90_
Polymyxin B and minocycline	0.25–4	1.0281	1	2
	0.25–8	2.2974	2	8
Polymyxin B and fosfomycin	0.25–4	1.3195	2	4
	64–1024	484.381	512	1024
Minocycline and fosfomycin	0.5–4	2.1140	2	4
	64–1024	526.39	512	1024

MIC_range_, the range of the MIC values; MIC_G_, the mean MIC value; MIC_50_, the MIC value at which 50% of bacteria were inhibited; MIC_90_, the MIC value at which 90% of bacteria were inhibited. The MIC values of the three drugs when used alone were significantly different from those when used in combination (P<0.05).

**Table III:** Fractional inhibitory concentration index (FICI) values of fosfomycin, minocycline and polymyxin B upon combined use.

Combined medication	FICI (≤0.5)	FICI (0.5–1)	FICI (1–4)	FICI (>4)
Polymyxin B and minocycline	44%	48%	8%	0
Polymyxin B and fosfomycin	16%	44%	40%	0
Minocycline and fosfomycin	12%	56%	32%	0
